# Supplementary material for: Reconciling Mining with the Conservation of Cave Biodiversity: A Quantitative Baseline to Help Establish Conservation Priorities
Source: PLoS One. 2016 Dec 20;11(12):e0168348. doi: 10.1371/journal.pone.0168348 (PMC5173368; doi:10.1371/journal.pone.0168348)
Supplement: S1 Dataset — (ZIP) [file pone.0168348.s002.zip › Taxa/Serra Norte/SN_2013/N4E/Lista N4E-62.pdf]

CAVIDADE N4E-0062

Classe	Ordem	Fam/Outros	Gên/Outros	Espécie	S-U	U-S	Zon.
Annelida	Oligochaeta			jovem		3	E
Arachnida	Acari	Laelapidae	*Cosmolaelaps*	sp.1	X		P/A
Arachnida	Acari	Prostigmata		sp.49	X		P
Arachnida	Amblypygi	Charinidae	*Charinus*	sp.1	1	14	E/P/A
Arachnida	Amblypygi	Phrynidae	*Heterophrynus*	*longicornis*	3	1	P/A
Arachnida	Araneae	Caponiidae		sp.1		X	A
Arachnida	Araneae	Drymusidae	*Drymusa*	*spelunca*		X	A
Arachnida	Araneae	Mysmenidae	*Microdipoena*	sp.1		X	E
Arachnida	Araneae	Ochyroceratidae	*Ochyrocera*	sp.1	X		P
Arachnida	Araneae	Ochyroceratidae	*Ochyrocera*	sp.2	X		A
Arachnida	Araneae	Ochyroceratidae	*Speocera*	sp.1	X	X	P/A
Arachnida	Araneae	Pholcidae	*Leptopholcus*	sp.1	X		E
Arachnida	Araneae	Pholcidae	*Mesabolivar*	*aurantiacus*	X		E/P
Arachnida	Araneae	Pholcidae	*Mesabolivar*	sp.1	X	X	A
Arachnida	Araneae	Theridiosomatidae	*Plato*	sp.1	X	X	P
Arachnida	Opiliones	Cosmetidae	*Roquettea*	*singularis*	1		E
Arachnida	Opiliones	Escadabiidae		sp.2	X		P/A
Arachnida	Opiliones	Sclerosomatidae	*Prionostema*	sp.1		1	E
Arachnida	Opiliones	Stygnidae	*Paraphareus*	sp.1		X	P
Arachnida	Pseudoscorpiones	Bochicidae		sp.1		X	A
Arachnida	Pseudoscorpiones	Chthoniidae	*Pseudochthonius*	sp.1	X		E/A
Arachnida	Schizomida	Hubbardiidae	aff. *Rowlandius*	sp.1	1		P/A
Arachnida	Scorpiones	Buthidae	*Ananteris*	jovem		1	P
Chilopoda	Scolopendromorpha	Cryptopidae	*Cryptops*	jovem	1	2	A
Chilopoda	Scolopendromorpha	Scolopocryptopidae		sp.1		1	A
Diplopoda	Polydesmida	Chelodesmidae		sp.3	1		P
Diplopoda	Polydesmida	Cyrtodesmidae		jovem	X		P
Diplopoda	Polydesmida	Cyrtodesmidae		sp.1	X		P
Diplopoda	Polydesmida	Fuhrmannodesmidae		sp.1	X	X	P/A
Diplopoda	Polydesmida	Pyrgodesmidae		sp.1	X	X	P
Entognatha	Collembola	Cyphoderidae		sp.1		X	E/P/A
Entognatha	Collembola	Paronellidae		sp.1	X		E/P
Entognatha	Collembola	Paronellidae		sp.4	X	X	P/A
Entognatha	Diplura	Campodeidae		sp.1	X		E/A
Insecta	Blattodea	Blattellidae		jovem	2	4	E/P
Insecta	Coleoptera	Carabidae		sp.1	X		P
Insecta	Coleoptera	Dytiscidae		sp.16	X		P
Insecta	Diptera	Ceratopogonidae		sp.	X		E
Insecta	Diptera	Culicidae	*Anopheles*	sp.		X	E
Insecta	Diptera	Culicidae	*Culex*	sp.	X	X	E/P
Insecta	Diptera	Dolichopodidae		sp.	X		E
Insecta	Diptera	Psychodidae	Phlebotominae	sp.	X	X	E/P/A
Insecta	Diptera	Streblidae	*Trichobius*	sp.	X	X	P
Insecta	Diptera	Tipulidae	Tipulinae	sp.	X	X	E
Insecta	Heteroptera	Reduviidae	Triatominae	sp.	2		A
Insecta	Heteroptera	Tingidae		sp.	X		A
Insecta	Heteroptera	Veliidae	*Paravelia*	sp.	X	X	E/P
Insecta	Homoptera	Cixiidae		sp.1	X	X	A
Insecta	Hymenoptera	Braconidae		sp.2		X	P

Insecta	Hymenoptera	Formicidae	*Paratrechina*	sp.1	X		P
Insecta	Hymenoptera	Formicidae	*Pheidole*	sp.1		X	A
Insecta	Hymenoptera	Formicidae	*Pheidole*	sp.3		X	A
Insecta	Hymenoptera	Formicidae	*Camponotus*	sp.2		X	E
Insecta	Hymenoptera	Formicidae	*Gigantiops*	sp.1	X		E
Insecta	Hymenoptera	Formicidae	*Hypoponera*	sp.1	X		P
Insecta	Hymenoptera	Formicidae	*Labidus*	sp.1		X	A
Insecta	Hymenoptera	Formicidae	Myrmicinae	sp.1	X		A
Insecta	Hymenoptera	Formicidae	*Odontomachus*	sp.1		X	E
Insecta	Hymenoptera	Formicidae	*Paratrechina*	sp.1	X	X	E/P/A
Insecta	Hymenoptera	Formicidae	*Paratrechina*	sp.2	X		P/A
Insecta	Hymenoptera	Formicidae	*Pheidole*	sp.2	X		E
Insecta	Hymenoptera	Formicidae	*Pheidole*	sp.3		X	E/A
Insecta	Isoptera	Termitidae	*Nasutitermes*	sp.	X	X	E/P/A
Insecta	Lepidoptera	Noctuoidea		sp.5	X	2	E/P
Insecta	Orthoptera	Gryllidae	Nemobiinae	sp.		2	A
Insecta	Orthoptera	Phalangopsidae	*Phalangopsis*	sp.	32	83	E/P/A
Insecta	Psocoptera			jovem	X		E
Insecta	Thysanura	Ateluridae		jovem	X		P
Gastropoda	Pulmonata	Bulimulidae	*Naesiotus*	sp.	X	X	P/A
Malacostraca	Decapoda	Pseudothelphusidae		sp.1		1	E
Malacostraca	Isopoda	Armadillidae		sp.1		X	P
Malacostraca	Isopoda	Platyarthridae		sp.1	X	X	P
Malacostraca	Isopoda	Scleropactidae	*Circoniscus*	*buckupi*		X	P/A
Symphyla		Scutigerellidae		sp.	X	X	P/A
Turbellaria	Tricladida			sp.2		5	P
Amphibia	Anura	Leptodactylidae	*Leptodactylus*	cf. *vastus*	1		P
Amphibia	Anura	Strabomantidae	*Pristimantis*	cf. *fenestratus*	2		P
Amphibia	Anura			sp.		2	P
Mammalia	Chiroptera	Emballonuridae	*Peropteryx*	*kappleri*	1	10	E/P/A
Mammalia	Chiroptera	Furipteridae	*Furipterus*	*horrens*	10	15	P/A
Mammalia	Chiroptera	Phyllostomidae	*Carollia*	*perspicillata*	16	10	P/A
Mammalia	Chiroptera	Phyllostomidae	*Glossophaga*	*soricina*	1	5	P/A
